# Inclusion of hybrid nanoparticles in hyperbolic tangent material to explore thermal transportation via finite element approach engaging Cattaneo-Christov heat flux

**DOI:** 10.1371/journal.pone.0256302

**Published:** 2021-08-25

**Authors:** Umar Nazir, Muhammad Sohail, Hussam Alrabaiah, Mahmoud M. Selim, Phatiphat Thounthong, Choonkil Park

**Affiliations:** 1 Department of Applied Mathematics and Statistics, Institute of Space Technology, Islamabad, Pakistan; 2 College of Engineering, Al Ain University, Al Ain, United Arab Emirates; 3 Department of Mathematics, Tafila Technical University, Tafila, Jordan; 4 Department of Mathematics, Al-Aflaj College of Science and Humanities Studies, Prince Sattam Bin Abdulaziz University, Al-Aflaj, Saudi Arabia; 5 Department of Mathematics, Suez Faculty of Science, Suez University, Suez, Egypt; 6 Research Institute for Natural Sciences, Hanyang University, Seoul, Korea; 7 Renewable Energy Research Centre, Department of Teacher Training in Electrical Engineering, Faculty of Technical Education, King Mongkut’s University of Technology North Bangkok, Bangkok, Thailand; Central University of Karnataka, INDIA

## Abstract

This report is prepared to examine the heat transport in stagnation point mixed convective hyperbolic tangent material flow past over a linear heated stretching sheet in the presence of magnetic dipole. Phenomenon of thermal transmission plays a vital role in several industrial manufacturing processes. Heat generation is along with thermal relaxation due to Cattaneo-Christov flux is engaged while modeling the energy equation. In order to improve the thermal performance, inclusion of hybrid nanoparticles is mixed in hyperbolic tangent liquid. The conservation laws are modeled in Cartesian coordinate system and simplified via boundary layer approximation. The modeled partial differential equations (PDEs) system are converted into ordinary differential equations (ODEs) system by engaging the scaling group transformation. The converted system of modeled equations has been tackled via finite element procedure (FEP). The efficiency of used scheme has been presented by establishing the grid independent survey. Moreover, accurateness of results is shown with the help of comparative study. It is worth mentioning that the inclusion of hybrid nanoparticles has significant higher impact on heat conduction as compared with nanoparticle. Moreover, hybrid nanoparticles are more efficient to conduct maximum production of heat energy as compared with the production of heat energy of nanoparticles. Hence, hybrid nanoparticles (*MoS*_2_/*Ag*) are observed more significant to conduct more heat energy rather than nanoparticle (*Ag*).

## 1. Introduction

Non-Newtonian fluids have diverse applications and usage in several engineering disciplines. There constitutive relation is different than Newtonian materials. For non-Newtonian materials, the stress strain relationship is complicated. These materials occur frequently in different phenomenon and it has applications in polymers extrusion, medical, oil-pipeline friction reduction and several others. An important non-Newtonian model is hyperbolic tangent [1–5], whose constitutive expression is expressed as

$$S^{*}=-PI+\tau_{TH}^{*},\tau_{TH}^{*}=-\dot{\alpha }[\mu_{\infty }+(\mu_{\infty }+\mu_{0})\tanh{\left(\Gamma^{1}\dot{\alpha }\right)}^{m}],$$

for $\mu_{\infty }=0,\Gamma^{1}\dot{\alpha }<1,$ we get

$$\tau_{TH}^{*}={-\dot{\alpha }\mu }_{0}[1+m(\dot{\alpha }-1)].$$

Naseer et al. [1] examined the exponentially stretched flow of mixed convective hyperbolic tangent model in horizontal cylinder with thermal transportation. They engaged shooting approach to solve transformed nonlinear boundary layer equations (BLEs). They have discussed the contribution of several influential variables on velocity and temperature. They observed the depreciation in temperature field for higher values of Reynolds number and Prandtl number. Khan et al. [2] used BVPh2.0 package to handle the solution for chemically reactive hyperbolic tangent model undergoing radiation and heat generation phenomenon. They have found that velocity is a diminishing function of Weissenberg number and temperature profile grows against radiation parameter. Nawaz et al. [3] explored the contribution of hybrid nanoparticles in hyperbolic tangent model in a stretched cylinder using finite element approach. They modeled the thermal phenomenon under radiation effect and heat generation. They observed that heat transfer rate of hybrid nanoparticles is higher than nanofluid and it is recommended for better thermal performance. Mathematical analysis for inclined MHD hyperbolic tangent model was studied by Ali et al. [4] via BVP4C package. They recorded the increase in Nusselt number for Prandtl number. Patil and Raju. [5] used the convective conditions in hyperbolic tangent model past over a nonlinear exponential porous stretching sheet. They modeled the energy transport in the presence of viscous dissipation and radiation effect. They used shooting approach to compute solution for the arising boundary layer equations. They concluded that higher values of Eckert number reduce the skin friction and enhances the Sherwood number.

Nanofluids are a hot topic of research due to their wider use in different industrial mechanisms. It is used in several engineering systems to control/enhance the thermal performance. The inclusion of nanoparticles in base fluids enhances the thermal conductivity which enhance in thermal process. Several researchers have a look on this hot topic due to their wider applications and usage. For instance, Aman et al. [6] studied the thermal performance of unsteady convective Casson model by mixing SWCNTs and MWCNTs in a vertical channel. They used the thermal conductivity model which was proposed by Maxwell. They have considered radiation effect in thermal profile and mixed convection along with MHD effect is taken in momentum transport. They presented the perturbation solution for the arising problem. Several graphs and tables are presented by them to notice the influence of emerging parameters on obtained solution. They reported the increase in thermal field by enhancing the values of radiation parameter and volume fraction because it upsurges the conduction. Moreover, they recorded the enhancement in velocity against Richardson parameter. Mixed convective stratified unsteady electrically conducting viscous nanofluid was investigated by Daniel et al. [7]. They modeled a strong problem over a melting stretching sheet with several important physical effects. They modeled a strong problem by taking several important effects like mixed convection, viscous dissipation, chemical reaction, radiation and heat generation which are used in the modeled laws. They presented the numerical solution via finite difference technique and established a comparative analysis to judge the scheme efficiency and results validity. They have shown an increase in momentum and thermal profile against electric parameter. Besthapu et al. [8] worked on thermally stratified dissipated stretched nanofluid past over a heating stretching porous sheet numerically. They plotted the solution against different physical parameters and noticed the decline in fluid velocity against magnetic parameter and increase in concentration and thermal fields. Also, they reported the rise in concentration and temperature profiles against mounting values of thermophoresis parameter. Ramzan et al. [9] discussed the optimal solution foe Maxwell nanofluid model with thermal and mass transportation having soret and dufour effects past over a porous heated sheet. They presented the error analysis graphically against different approximations order. They noticed the diminishing impact of porous parameter on velocity. Also, an enhancement in heat and mass transfer coefficients is monitored by them against suction parameter and viscoelastic parameter. Modelling of water based nanoparticles in rotating disc having Darcy Forchhiemer medium with slip effects was analyzed by Hayat et al. [10] analytically. They have mixed different type of nanoparticles in water to investigate thermal performance. They used the relation proposed by Xue for thermos-physical properties. They monitored the enhancement in heat transfer coefficient against Forchhiemer number and retardation in fluid velocity for higher volume fraction. Khanafer and Vafai [11] deliberated the experimental analysis for thermophysical features of nanofluids. They have critically studied different effective viscosity models and thermal expansion coefficient in their reported study. Kempannagari et al. [12] discussed characteristics of heat transfer in micropolar liquid including the effects of thermal conductivity (variable), thermal radiation and Joule heating past a heated curved sirafce. Kumar et al. [13] discussed the aspects of thermal energy in hybrid ferrofluid considering the impacts of thermal radiation and heat generation. Kumar et al. [14] addressed the phenomenal contrast in view of heat transfer and flow behavior over wedge and heated cone. They considered the theory of non-Fourier’s under the action of magnetic field via shooting approach. They also investigated that flow phenomena and heat transfer for the case of cone are higher than flow phenomena and heat transfer for the case of wedge. Kumar et al. [15] studied influences of thermal energy along with thermal radiation in the attendance of magnetic field past a heated curved surface via shooting scheme. Kumar et al. [16] captured the behavior of micropolar fluid including the BCs (2^nd^ order and first order slips) whereas they considered variable heat (sink) and Lorentz force. Kumar et al. [17] investigated the flow situation in the attendance of magnetic field. They used slandering heated surface to know the behavior considering effects along with viscous dissipation. Anantha Kumar et al. [18] observed rheology of micropolar fluid along with stagnation point and magnetic field. They used second-order (velocity slip) of considered flow model past a heated vertical surface. They also considered the thermal conductivity (variable), Joule heating and heat source sink and electrical energy. Ashwinkumar [19] visualized the influences of thermal energy and solute particles along with action of magnetic field inserting nanoparticles (alloy/silver‐water) past an elongated heated surface. Ashwinkumar and Sulochana et al. [20] simulated consequences buoyancy force and thermal radiation under the action of magnetic field inserting *Al*_50_*Cu*_50_-nanoparticles in the rheology of Casson fluid. Ashwinkumar et al. [21] securitized features of ferrous nanoparticles under the action of magnetic field over melting vertical plate (semi-infinite). Sulochana et al. [22] discussed the aspects of heat transfer phenomena in Maxwell liquid including the nanoparticles over a melting elongated surface. They considered various kinds of influences (heat generation, thermophoresis, Brownian moment and magnetic field) simulated the results via shooting scheme. Mabood et al. [23] discussed an enhancement in thermal energy inserting the hybrid nanoparticles (Fe3O4/graphene–*H*_2_0) in the attendance of magnetic field. Mabood et al. [24] simulated the maximum production of heat energy due the role hybrid nanoparticles under the action of thermal radiation and magnetic field past a slandering melting surface. They used shooting scheme to simulate the simulations of considered model. Samrat et al. [25] investigated the rheology of Casson liquid inserting nanoparticles in the presence of thermal rediation past a stretching surface. Sulochana et al. [26] discussed the phenomena of thermal energy in the rheology of ferrofluid involvement of nanoparticles towards the horizontal needle via magnetic field. They studied the role of ferrous nanoparticles in solute particles and thermal energy including magnetic field and thermal radiation subjected to chemical reaction over an elongated plate. Sulochana et al. [27] studied the impact of 2D flow, thermal energy and mass diffusion inserting nanoparticles under action of thermal radiation and chemical reaction over a heated elongated plate via shooting scheme. Some important contributions are reported in [28–36].

**Fig 1:**
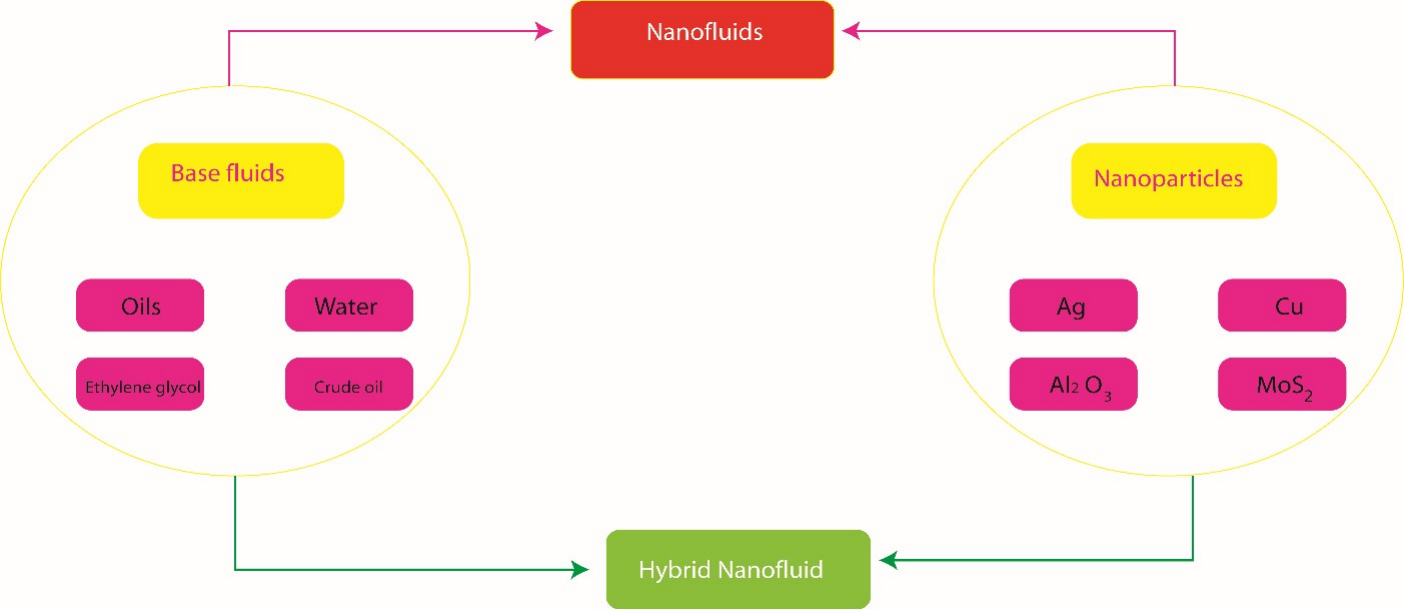
Manufacturing approach of hybrid nanoparticles and nanoparticles.

Above cited studies ensure that no analysis has been done for hyperbolic tangent model with thermal relaxation time and radiation phenomenon. This contribution fills this gap. This contribution is organized as: section 1 consists of literature survey, modeling of considered flow situation with physical quantities are listed in section 2, solution methodology with advantages is reported in section 3, graphical and tabular results are analyzed in section 4 and section 5 contains the important findings. The sketched scheme of hybrid nanoparticles and nanoparticles are illustrated by Fig 1.

### 2. Modeling development

**Fig 2:**
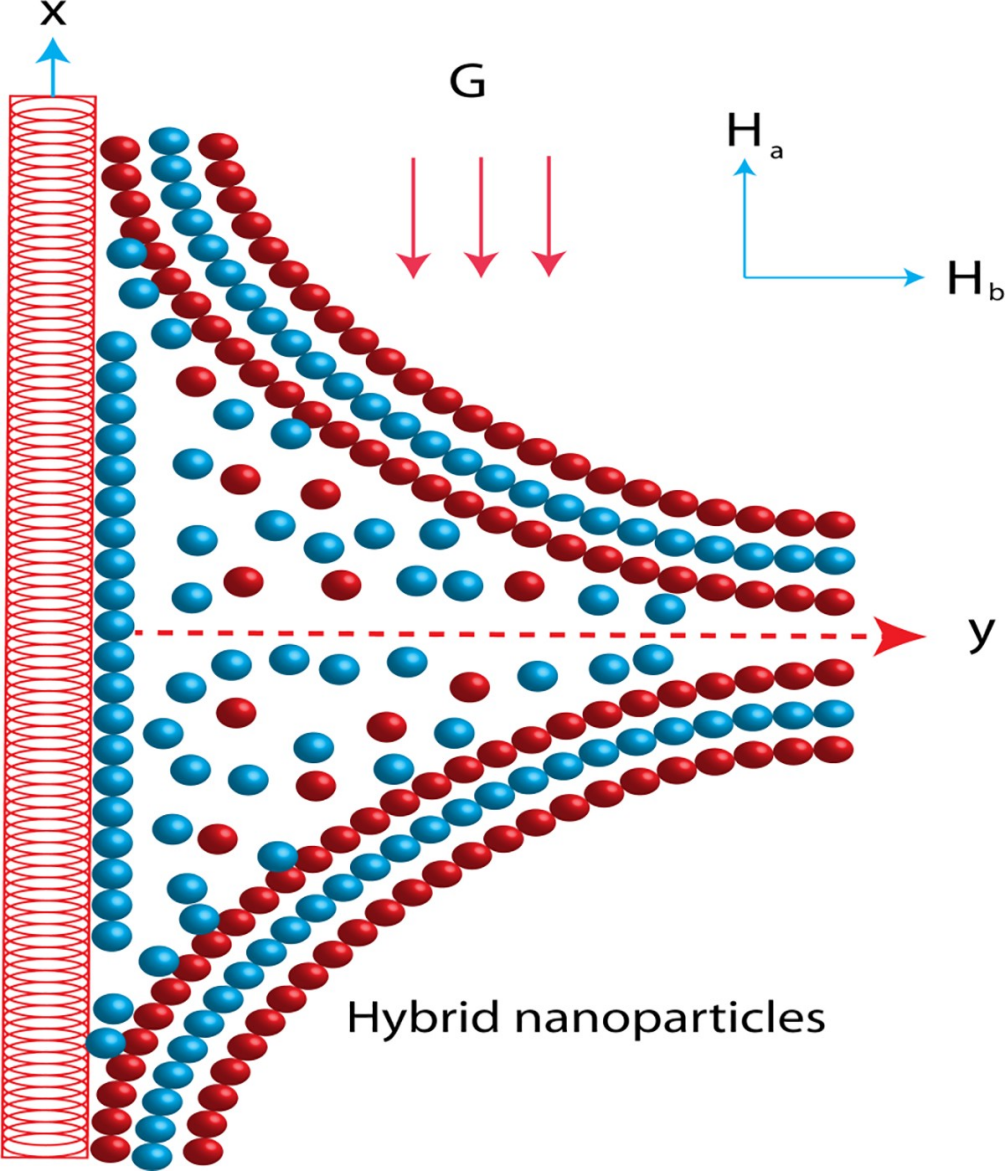
Geometrical view of flow model.

The modeling of 2D hyperbolic tangent liquid [1–5] inserting the thermal properties of nano and hybrid nanostructures is addressed past a melting surface. The non-Fourier’s law [14, 36] in the presence of heat generation is studied in energy equation. The movement in fluid particles is responsible due to velocity (*bx*) of surface. The role of magnetic induction is captured and magnetic field is also taken at away from the surface considered by *H*_0_*x*. The base fluid is considered as a EG (ethylene glycol) along with hyperbolic tangent liquid while nanoparticles (*Ag*) and hybrid nanoparticles are called *MoS*_2_/*Ag*. The geometrical phenomenon is demonstrated by Fig 2. The considerations of current model are addressed below:

➢ Two dimensional flow in the presence of hyperbolic tangent liquid is considered;
➢ Cattaneo-Christove heat flux is addressed;
➢ The role of heat generation is assumed;
➢ Steady flow is taken out;
➢ The behavior of magnetic induction is captured;
➢ Hybrid nanoparticles and nanoparticles are inserted;
➢ Thermal properties and correlations of *Ag* and *MoS*_2_/*Ag* in ethylene glycol;
➢ 2^nd^ law analysis associated with mixed convection is observed;
➢ The vertical melting surface is considered.

The set of PDEs [35] are generated using (BLA) boundary layer approximations which are

$$\frac{\partial \tilde{U}}{\partial x}+\frac{\partial \tilde{V}}{\partial y}=0,$$ (1)

$$\frac{\partial H_{a}}{\partial x}+\frac{\partial H_{b}}{\partial y}=0,$$ (2)

$$\tilde{U}\frac{\partial \tilde{U}}{\partial x}+\tilde{V}\frac{\partial \tilde{U}}{\partial y}-\frac{\tilde{\mu }}{4(\pi )\rho_{f}}\left(H_{a}\frac{\partial H_{a}}{\partial x}+H_{b}\frac{\partial H_{b}}{\partial y}\right)=\frac{\mu_{f}}{A_{2}A_{1}}\left[(1-m)\frac{\partial^{2}\tilde{U}}{\partial y^{2}}+m\sqrt{2}\Gamma^{1}(\frac{\partial \tilde{U}}{\partial y})\frac{\partial^{2}\tilde{U}}{\partial y^{2}}\right]$$ (3)

$$+G\frac{{\left(\rho \beta \right)}_{f}}{\rho_{f}}A_{5}(T-T_{\infty })-\frac{\tilde{\mu }}{4(\pi )\rho_{f}}H_{e}\left(\frac{dH_{e}}{dx}\right)+U_{\infty }\frac{dU_{\infty }}{dx},$$ (4)

$$\tilde{U}\frac{\partial H_{a}}{\partial x}+\tilde{V}\frac{\partial H_{b}}{\partial y}=\mu_{e}\frac{\partial^{2}H_{a}}{\partial y^{2}}+H_{a}\frac{\partial \tilde{U}}{\partial x}+H_{b}\frac{\partial \tilde{U}}{\partial y},$$ (5)

$$\begin{array}{c} \tilde{U}\frac{\partial T}{\partial x}+\tilde{V}\frac{\partial T}{\partial y}+\lambda_{A}\left(\begin{array}{c} \tilde{U}\frac{\partial \tilde{U}}{\partial x}\frac{\partial T}{\partial x}+\tilde{V}\frac{\partial \tilde{V}}{\partial y}\frac{\partial T}{\partial x}+\tilde{U}\frac{\partial \tilde{V}}{\partial x}\frac{\partial T}{\partial y}+2\tilde{U}\tilde{V}\frac{\partial^{2}T}{\partial x\partial y}\\ {\tilde{U}}^{2}\frac{\partial^{2}T}{\partial x^{2}}+{\tilde{V}}^{2}\frac{\partial^{2}T}{\partial y^{2}}-\frac{Q}{{\left(\rho C_{p}\right)}_{hnf}}(\tilde{U}\frac{\partial T}{\partial x}+\tilde{V}\frac{\partial T}{\partial y}) \end{array}\right)\\ =\frac{k_{hnf}}{{\left(\rho C_{p}\right)}_{hnf}}\frac{\partial^{2}T}{\partial^{2}y}+Q(T-T_{\infty }). \end{array}$$ (6)

Where $\tilde{U},\tilde{V}$ (velocity components), *x*,*y* (space coordinates), *m* (power law index number), $\tilde{H}$ (induced magnetic number), *H*_*a*_, *H*_*b*_ (components of magnetic induction), $\tilde{\mu }$ (magnetic permeability), *μ*_*e*_ (magnetic diffusivity), *T* (fluid temperature), *ρ* (fluid density), *T*_∞_ (ambient temperature), *Q* (heat generation), *k* (thermal conductivity), *λ*_*A*_ (relaxation number), *G* (gravitation force), *μ*_*f*_ (fluid dynamic viscosity), Γ^1^ (time constant number) and *β* (thermal expansion) are mentioned above.

No-slip theory provides required boundary conditions (BCs) are

$$\begin{array}{c} \tilde{U}=cx=U_{w},\tilde{V}=0,T=T_{w},\frac{\partial H_{a}}{\partial y}=0,H_{b}=0,y=0,\\ \tilde{U}\to (ax),T=T_{\infty },H_{a}\to {xH}_{0},y\to \infty . \end{array}$$ (7)

The variables are used to obtain ODEs and these variables are:

$$\begin{array}{c} \tilde{U}=cxf_{\eta },\tilde{V}=-{\left(c\nu_{f}\right)}^{\frac{1}{2}}f,\eta =y{\left(\frac{c}{\nu_{f}}\right)}^{\frac{1}{2}},\\ H_{a}=H_{0}xg_{\eta },H_{b}=-{\left(c\nu_{f}\right)}^{\frac{1}{2}}g,\theta (T_{w}-T_{\infty })=T-T_{\infty }, \end{array}$$ (8)

The system of ODEs along with desired BCs are addressed [35] as

$$\begin{array}{c} {(1-m)f}_{\eta \eta \eta }+mWef_{\eta \eta }f_{\eta \eta \eta }-A_{1}A_{2}({f^{\prime }}^{2}-ff^{\prime \prime }-A^{2})+A_{1}A_{2}\beta ({g^{\prime }}^{2}-gg^{\prime \prime }-1)\\ +{A_{5}A}_{1}A_{2}\lambda_{1}\theta =0,\\ f(0)=0,f_{\eta }(0)=1,f_{\eta }(\infty )\to A, \end{array}\},$$ (9)

$$\begin{array}{c} g_{\eta \eta \eta }+\frac{1}{\lambda }(fg_{\eta \eta }-f_{\eta \eta }g)=0,\\ g(0)=0,g_{\eta \eta }(0)=1,g_{\eta }(\infty )\to 1 \end{array}\},$$ (10)

$$\begin{array}{c} \theta_{\eta \eta }+\frac{k_{f}{\left(\rho C_{p}\right)}_{hnf}}{k_{hnf}{\left(\rho C_{p}\right)}_{f}}Prf\theta_{\eta }-\frac{k_{f}{\left(\rho C_{p}\right)}_{hnf}}{k_{hnf}{\left(\rho C_{p}\right)}_{f}}Pr\Omega_{a}\left[ff_{\eta }\theta_{\eta }+f^{2}\theta_{\eta \eta }+H_{s}f\theta_{\eta }\right]\\ +\frac{k_{f}}{k_{hnf}}H_{s}Pr\theta =0,\\ \theta (0)=1,\theta (\infty )=0. \end{array}\}.$$ (11)

The correlations of thermo-physical properties in nano and hybrid nanoparticles are mentioned below and their values are listed in Table 1.

**Table 1:** Properties related to thermal of nanoparticles and hybrid nanoparticles in EG.

*MoS*_2_	*Ag*	*C*_2_ *H*_6_ *O*_2_
$\rho_{MoS_{2}}(=5060)$	*ρ*_*Ag*_( = 10490)	$\rho_{C_{2}H_{6}O_{2}}(=1113.5)$
${\left(C_{p}\right)}_{MoS_{2}}(=397.21)$	(*C*_*p*_)_*Ag*_( = 235)	${\left(C_{p}\right)}_{C_{2}H_{6}O_{2}}(=2430)$
$k_{MoS_{2}}(=904.4)$	*k*_*Ag*_(429)	$k_{C_{2}H_{6}O_{2}}(=0.253)$
$\beta_{MoS_{2}}(=2.8424\times 10^{-5})$	*β*_*Ag*_( = 1.89×10^−5^)	$\beta_{C_{2}H_{6}O_{2}}(=5.8\times 10^{-4})$
$\sigma_{MoS_{2}}(=2.09\times 10^{-5})$	*σ*_*Ag*_( = 6.30×10^7^)	$\sigma_{C_{2}H_{6}O_{2}}(=4.3\times 10^{-5})$


$$\begin{array}{c} \rho_{nf}=(1-\phi )\rho_{f}+\phi \rho_{s},\rho_{hnf}=[\left(1-\phi_{2})\{(1-\phi_{1})\rho_{f}+\phi_{1}{\rho_{s}}_{1}\right\}]+\phi_{2}{\rho_{s}}_{2},\\ {\left(\rho C_{p}\right)}_{nf}=(1-\phi ){\left(\rho C_{p}\right)}_{f}+\phi {\left(\rho C_{p}\right)}_{s},{\left(\rho C_{p}\right)}_{hnf}=[\left(1-\phi_{2})\{\begin{array}{c} (1-\phi_{1}){\left(\rho C_{p}\right)}_{f}\\ +\phi_{1}{{\left(\rho C_{p}\right)}_{s}}_{1} \end{array}\right\}]\\ {+\phi }_{1}{{\left(\rho C_{p}\right)}_{s}}_{2}, \end{array}\}$$ (12)

$$\begin{array}{c} \mu_{nf}=\frac{\mu_{f}}{{\left(1-\phi \right)}^{2.5}},\mu_{nf}=\frac{\mu_{f}}{{\left(1-\phi_{2}\right)}^{2.5}{\left(1-\phi_{1}\right)}^{2.5}},\frac{k_{nf}}{k_{f}}=\{\frac{k_{s}+(n+1)k_{f}-(n-1)\phi (k_{f}-k_{s})}{k_{s}+(n-1)k_{f}+\phi (k_{f}-k_{s})}\},\\ \frac{k_{hnf}}{k_{bf}}=\{\frac{k_{s_{2}}+(n-1)k_{bf}-(n-1)\phi_{2}(k_{bf}-k_{s_{2}})}{k_{s_{2}}+(n-1)k_{bf}-\phi_{2}(k_{bf}-k_{s_{2}})}\},\frac{\sigma_{hnf}}{\sigma_{f}}=(1+\frac{3(\sigma -1)\phi }{(\sigma +2)-(\sigma -1)\phi }),\\ \frac{\sigma_{hnf}}{\sigma_{f}}=(\frac{\sigma_{s_{2}}+2\sigma_{f}-2\phi_{2}(\sigma_{bf}-\sigma_{s_{2}})}{\sigma_{s_{2}}+2\sigma_{f}+\phi_{2}(\sigma_{bf}-\sigma_{s_{2}})}),\frac{\sigma_{bf}}{\sigma_{f}}=(\frac{\sigma_{s_{1}}+2\sigma_{f}-2\phi_{1}(\sigma_{f}-\sigma_{s_{1}})}{\sigma_{s_{1}}+2\sigma_{f}+\phi_{1}(\sigma_{f}-\sigma_{s_{1}})}) \end{array}\}$$ (13)

$$\begin{array}{c} A_{1}={\left(1-\phi_{2}\right)}^{\frac{5}{2}}{\left(1-\phi_{1}\right)}^{\frac{5}{2}},A_{2}=(1-\phi_{2})[1-\phi_{1}+\phi_{1}\frac{\rho_{s_{1}}}{\rho_{f}}]+\phi_{2}\frac{\rho_{s_{2}}}{\rho_{f}},\\ A_{3}=[1-\phi_{1}+\phi_{1}\frac{{\left(\rho c_{p}\right)}_{s_{1}}}{{\left(\rho c_{p}\right)}_{f}}]+\phi_{2}\frac{{\left(\rho c_{p}\right)}_{s_{2}}}{{\left(\rho c_{p}\right)}_{f}},\\ A_{5}=\frac{\phi_{2}\frac{{\left(\rho \beta \right)}_{s_{2}}}{{\left(\rho \beta \right)}_{f}}+[(1-\phi_{1}-\phi_{2})+\phi_{2}\frac{{\left(\rho \beta \right)}_{s_{2}}}{{\left(\rho \beta \right)}_{f}}]}{(1-\phi_{1})[(1-\phi_{1})+\phi_{2}\frac{\rho s_{1}}{\rho_{f}}]+\phi_{2}\frac{\rho s_{2}}{\rho_{f}}}. \end{array}\}$$ (14)

Here *ϕ*_2_, *ϕ*_1_, *ϕ* (volume fractions), *hnf* (hybrid nanoparticles) and *nf* (nanoparticles) are considered in Eqs (9–14). Further, *Pr* (Prandtl number), *λ* (magnetic Prandtl number in term of reciprocal), *β* (magnetic number), *A* (stretching ratio number), *H*_*s*_ (heat generation number), *λ*_1_ (mixed convection number), Ω_*a*_ (relaxation number) and *We* (Weissenberg number) are addressed as:

$$Pr={\left(\mu C_{p}\right)}_{f}{\left(k_{f}\right)}^{-1},\lambda =\frac{\mu_{e}}{\nu_{f}},\beta =\frac{\tilde{\mu }H_{0}^{2}}{{c^{2}\rho }_{f}4\pi },A=\frac{a}{c},$$

$$H_{s}=\frac{Q}{{c(\rho C_{p})}_{f}},\lambda_{1}=\frac{g\beta (T_{w}-T_{\infty })}{xc^{2}},\Omega_{a}={b\lambda }_{A},We={\left(\frac{c^{3}\Gamma x^{2}}{\nu_{f}}\right)}^{1/2}.$$

The divergent velocity at melting surface is derived as

$$C_{f}=\frac{\tau_{xy|y=0}}{\rho_{f}{\left(bx\right)}^{2}},\sqrt{R_{e}}C_{f}=\frac{-1}{{{\left(1-\phi_{2}\right)}^{5/2}(1-\phi_{1})}^{5/2}}\left[(1-m)f_{\eta \eta }(0)+\frac{m}{2}We{\left(f_{\eta \eta }(0)\right)}^{2}\right].$$

The temperature gradient of current phenomenon is

$$Nu=\frac{-xk_{hnf}\frac{\partial T}{\partial y}{|}_{y=0}}{{(T_{w}-T_{\infty })k}_{f}},{\left(R_{e}\right)}^{-1/2}Nu=-\frac{k_{hnf}}{k_{f}}\theta_{\eta }(0),$$

where (Reynolds number) $Re\left(=\frac{U_{w}x}{\nu_{f}}\right).$

## 3. Solution scheme and convergence analysis

The non-linear ODEs are numerically simulated using numerical scheme called finite element approach [33, 34]. Such numerical approach has ability to compute numerical solution of current complex problem. The main steps related this scheme is discussed here:

❖ The residuals (weighted integral) are made whereas the residuals are made using the integrating approach in set of non-linear ODEs (ordinary differential equations);
❖ In next step, computational domain is achieved through problem domain considering [0, ∞). It is demonstrated that numerical computations reveal that asymptotic BCs are agreed at *η*_∞_ = 7. Hence, [0, 7] is considered as computation domain;
❖ The stiffness matrix is computed using GFEA (Galerikin finite element approximation) in weak form of residuals (weighted integrals). The stiffness elements along with 300 elements in integral residuals are made. Finally, assembly scheme is captured;
❖ A Picard linearization scheme is utilized to make linearization in algebraic equations. The tolerance is captured as 10^−8^ for solving equations;
❖ The convergence approach is addressed considering 300 elements. Hence, current problem is converged at mid of each 300 elements. This convergence study is simulated in Table 2. Each outcome is simulated at the mid of each element. The validation of numerical results related to gradient temperature is simulated by Table 3.

**Table 2:** Convergence analysis of velocities and thermal energy considering 300 elements.

Number of elements	$f^{\prime }\left(\frac{\eta_{\infty }}{2}\right)$	$g^{\prime }\left(\frac{\eta_{\infty }}{2}\right)$	$\theta \left(\frac{\eta_{\infty }}{2}\right)$
30	0.4853074	0.5326158	0.5946054
60	0.4568591	0.5163933	0.5772373
90	0.4476232	0.5110348	0.5716849
120	0.4429796	0.5082616	0.5687690
150	0.4402813	0.5066645	0.5668638
180	0.4384072	0.5055560	0.5660132
210	0.4371181	0.5047064	0.5644715
240	0.4361482	0.5041676	0.5641853
270	0.4353591	0.5036810	0.5637239
300	0.4347759	0.5033553	0.5634322

**Table 3:** Comparison of simulations in view of Nusselt number when *ϕ*_1_ =0, *ϕ*_2_ = 0, *A* = 3.0, *β* = 1.

*Pr*	Iqbal et al. [35]	Present results
0.07	0.33814	0.3387103
0.5	0.82748	0.8273089
2.0	1.52147	1.5239940
6.8	2.59780	2.5971029
10.0	3.07902	3.0698597

## 4. Results and discussion

The code related to finite element approach called Galerikin finite element scheme is developed in Maple 18. This numerical approach has ability to simulate the complex flow problems. The current problem is addressed physical happening in view of PDEs including characterizations magnetic induction and non-Fourier’s theory. In this section, prime graphical outcomes of motion and thermal energy in fluid particles in the presence of heat enhancement role of nano and hybrid nanoparticles versus variation parameters are simulated. The prime discussion is addressed below:

## 4.1 Graphical outcomes of flow situation

**Fig 3:**
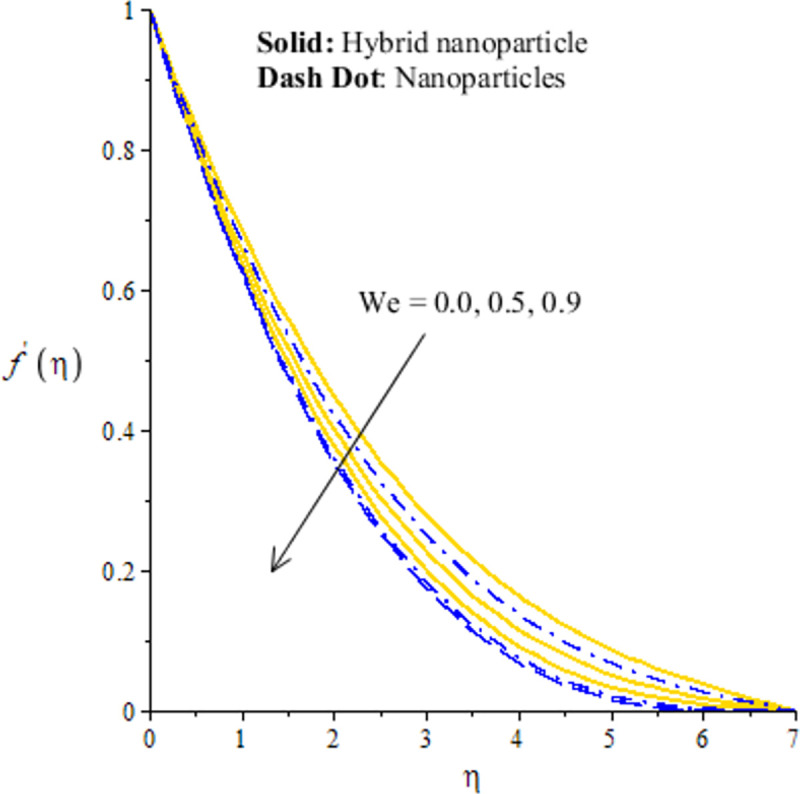
Distribution of velocity versus *we*.

**Fig 4:**
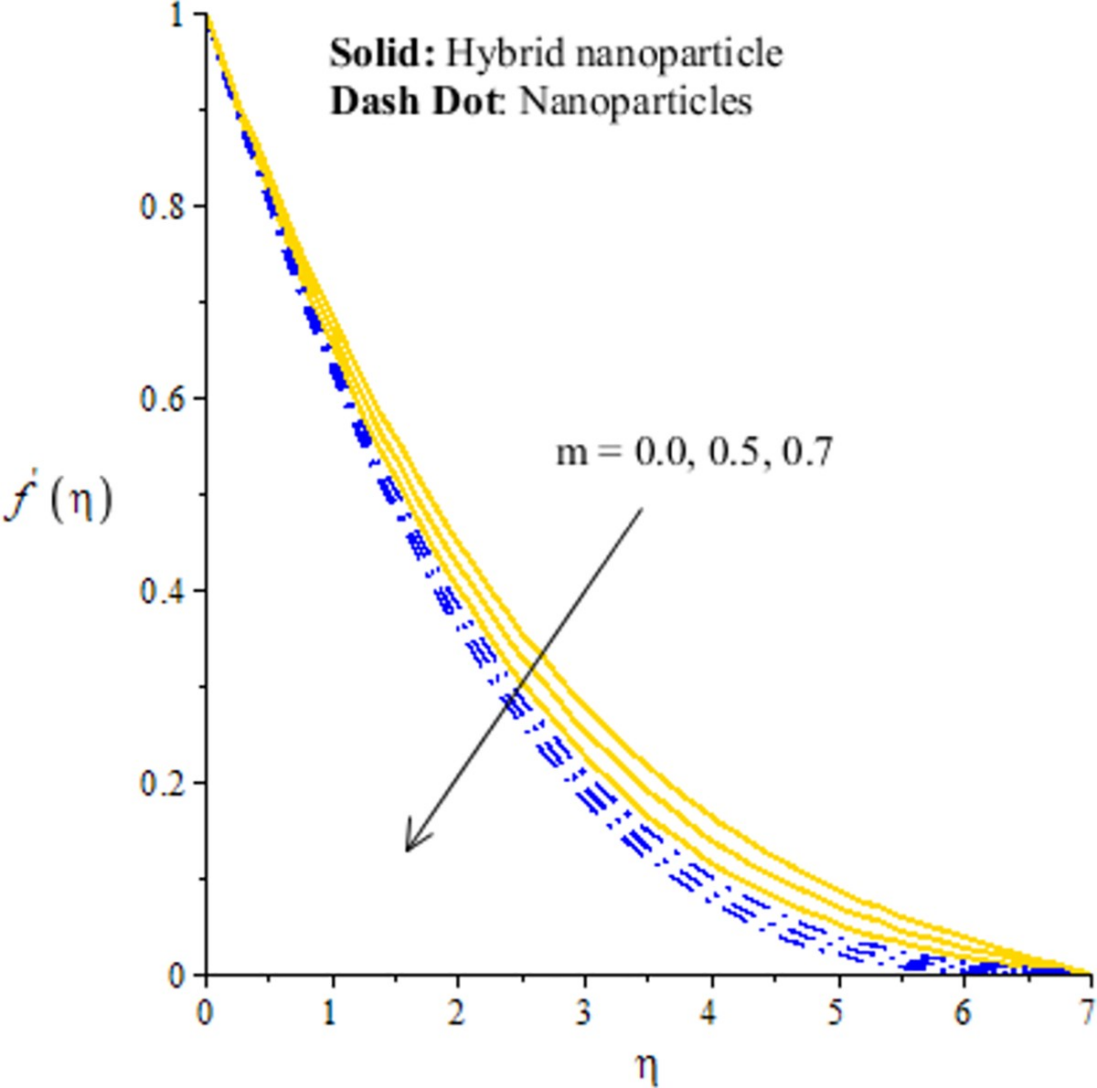
Distribution of velocity versus *m*.

**Fig 5:**
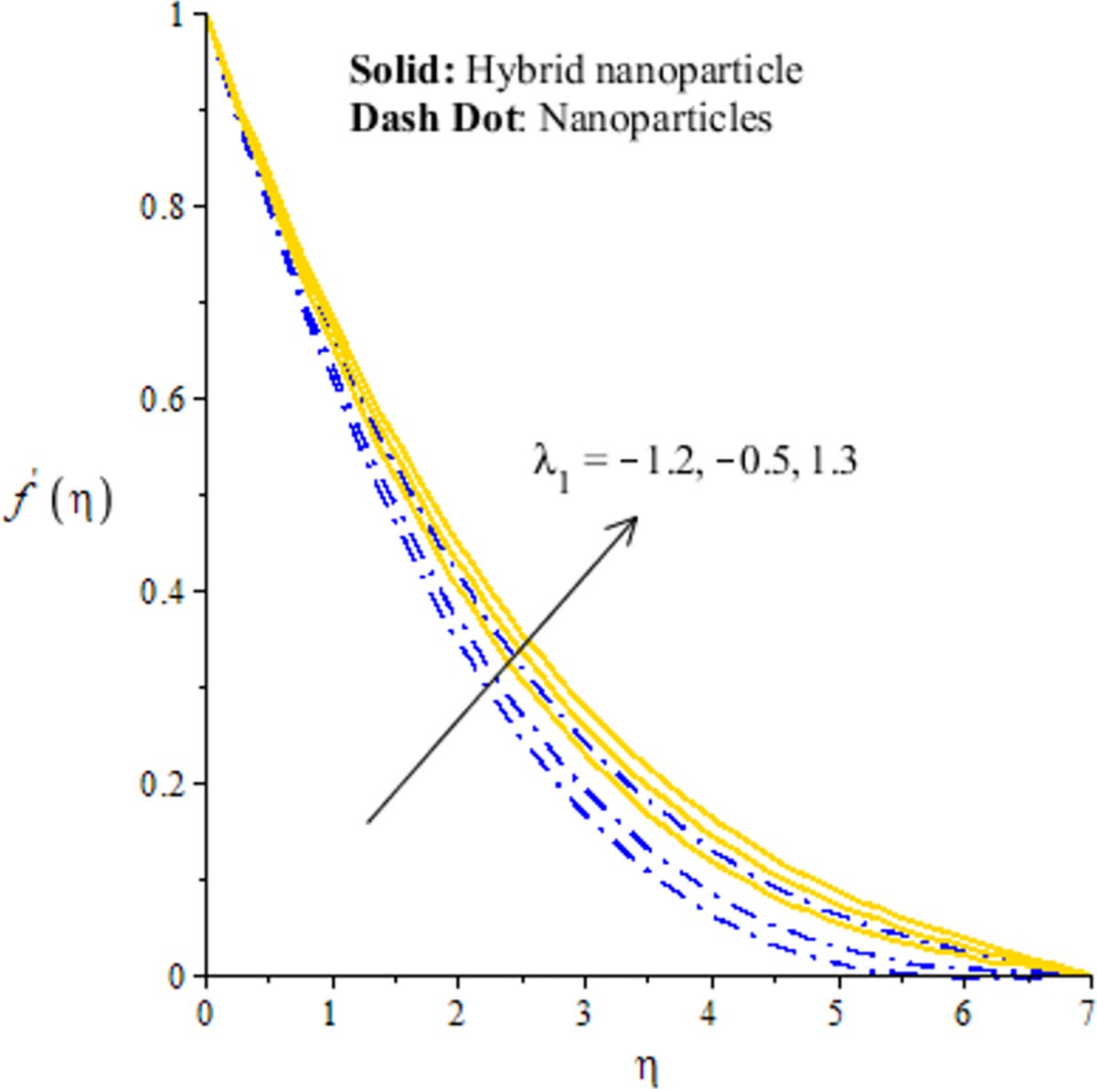
Distribution of velocity versus *λ*_1_.

**Fig 6:**
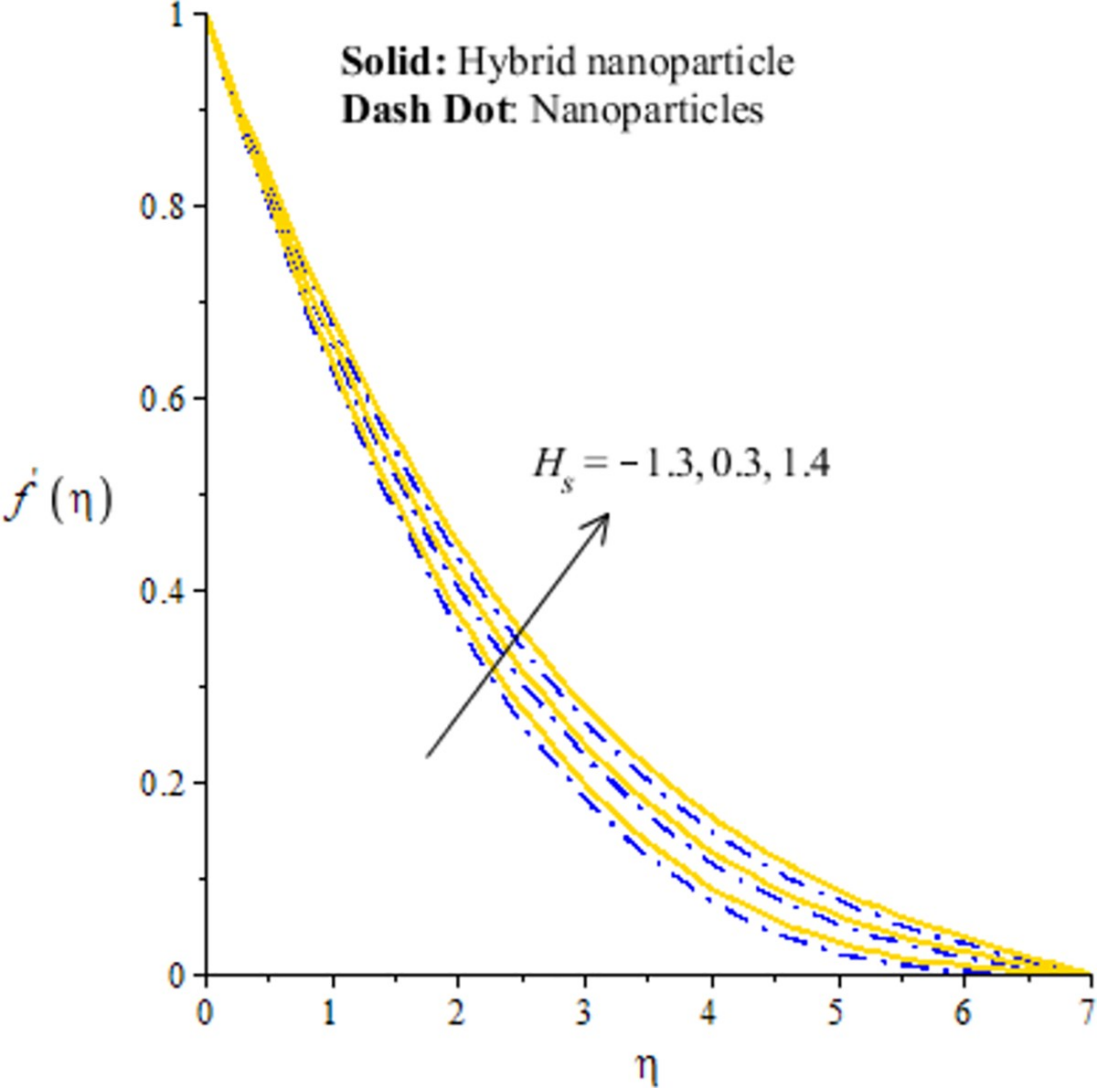
Distribution of velocity versus *H*_*s*_.

**Fig 7:**
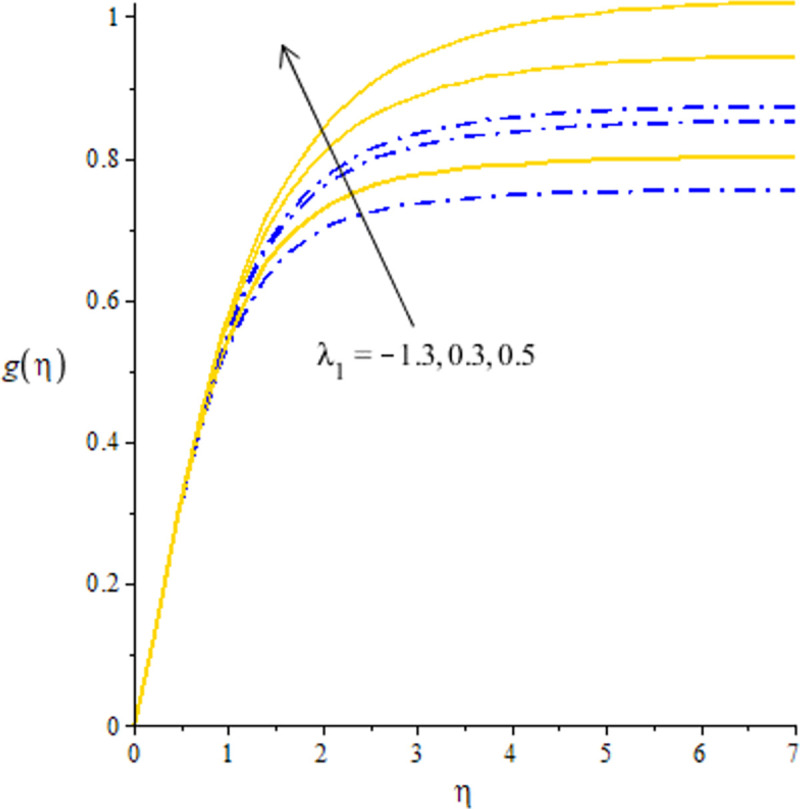
Distribution of velocity versus *λ*_1_.

**Fig 8:**
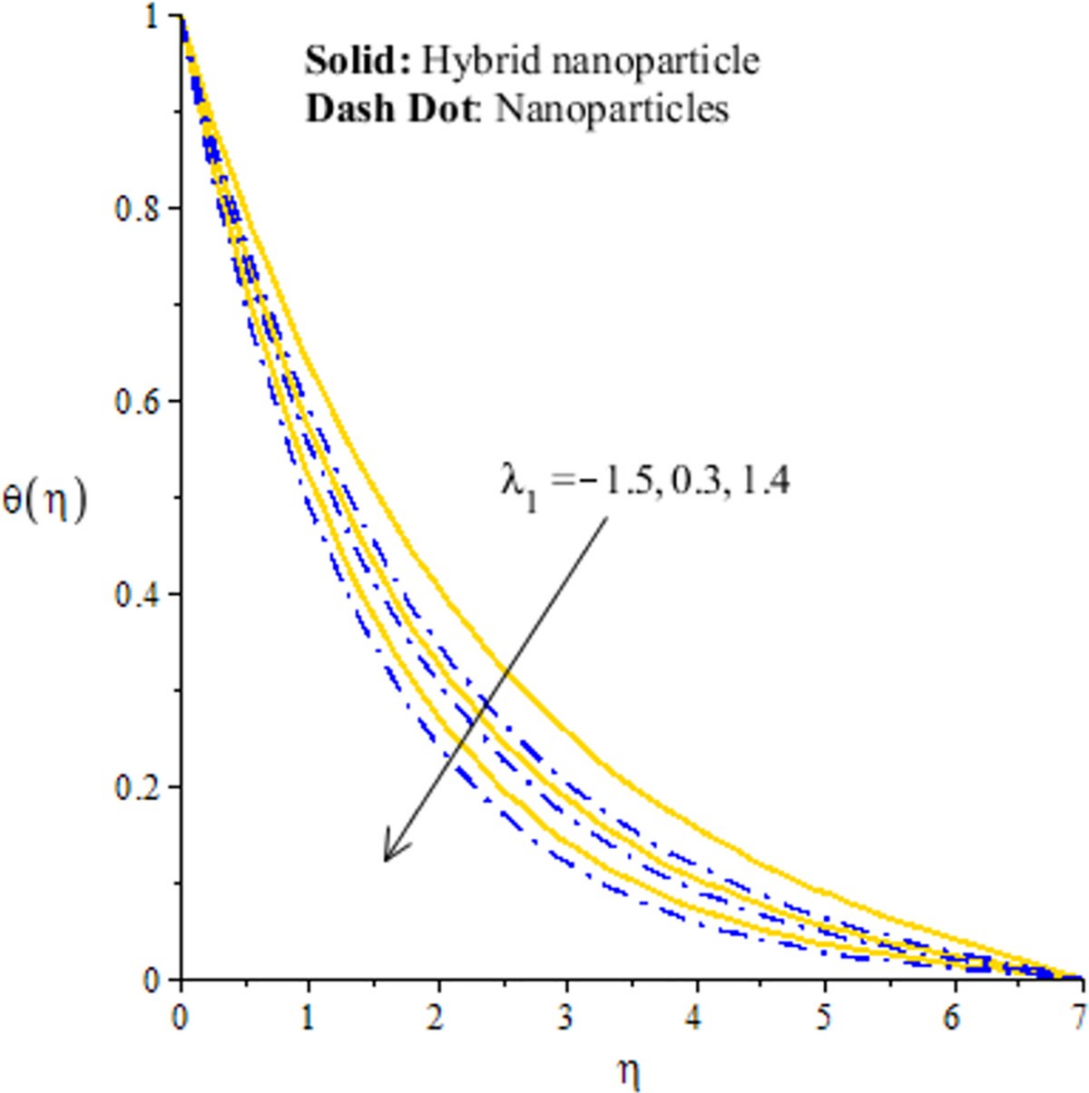
Distribution of temperature versus *λ*_1_.

**Fig 9:**
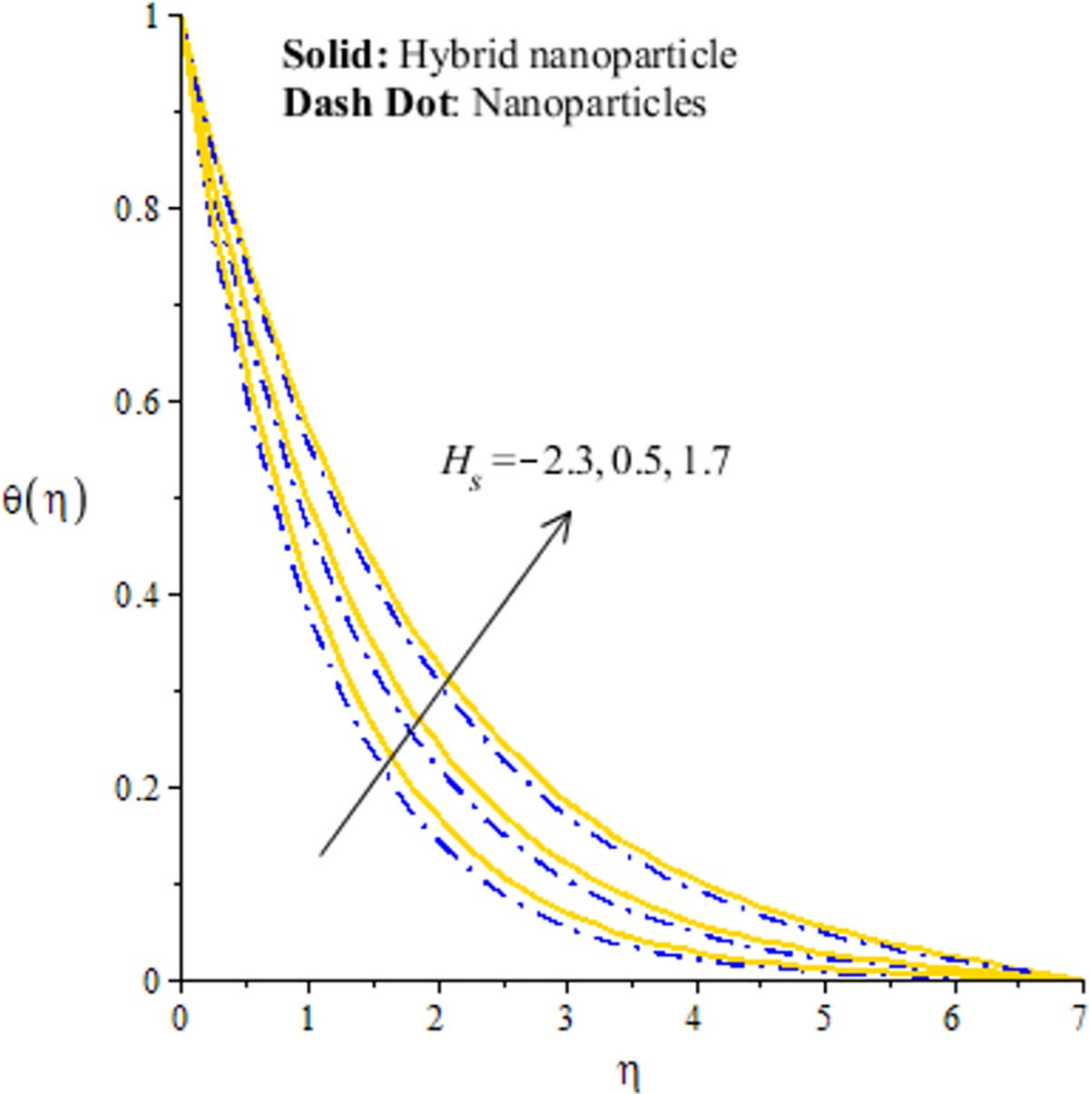
Distribution of temperature versus *H*_*s*_.

**Fig 10:**
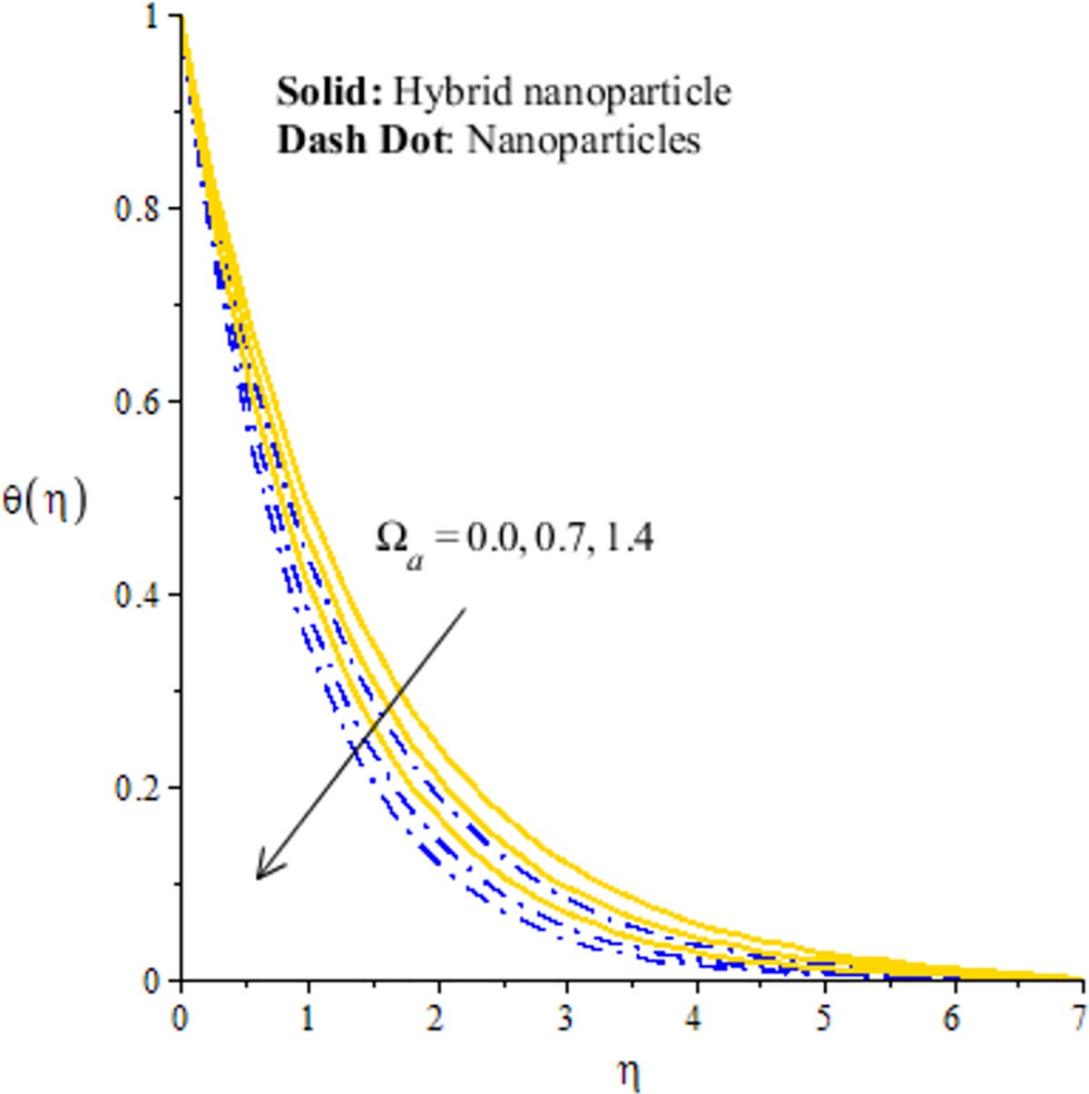
Distribution of temperature versus Ω_*a*_.

The measurement of flow situation is addressed versus the variation in *We* (Weissenberg number), *H*_*s*_ (heat generation number), *λ*_1_ (mixed convection number) and *m* (power law index number) inserting the nanoparticles and hybrid-nanostructures is captured. It is investigated that solid curves reveal that role of nanoparticles while dash dot lines are generated the influence of hybrid nanoparticles considering by Figs 3–10. Further, *Ag* is called nanoparticles and composite of *Ag* and *MoS*_2_ is known as hybrid nanoparticles. Fig 3 illustrates graphical variation in flow of nano and hybrid nanoparticles taking higher values of Weissenberg number. It is noticed that the concept of Weissenberg number is modeled due to the appearance of hyperbolic tangent tensor in momentum equation. Therefore, role of *We* is observed on the flow. In this figure, (*We*) generates the retardation force in motion of particles and this retardation force makes the reduction in flow speed of fluid particles. Physically, *We* is the ratio of viscous forces and elastic forces and viscous forces are increased during the flow of nanoparticles and hybrid nanoparticles versus the higher values of (*We*). So, higher viscous forces create the resistance force during flow of fluid particles when (*We*) is enhanced. Hybrid nanoparticles are more efficient in view of flow speed as compared nanoparticles. The distribution of flow speed is measured against the variation of power law index number (see Fig 4). The numerical values of *m* decides flow behavior whereas *m* is occurred due to tensor of hyperbolic tangent liquid. For *m* = 0, the fluid becomes Newtonian liquid and fluid becomes shear thickening using *m*<0. The flow becomes thick using large values of *m*. Therefore, *m* is not favorable as physical parameter to obtain the maximum flow speed. Hence, decreasing trend is measured against enlargement in *m*. From Fig 4, study of hybrid nanoparticles is favorable rather than nanoparticles in base fluid called ethylene glycol. Figs 5 and 7 demonstrate the flow situation versus the variation of *λ*_1_. The increment in flow behavior is found against the large value of *λ*_1_. This parameter generates maximum magnitude in flow and maximum flow speed is happened due to higher temperature gradient. The maximum convection and bouncy force are generated due to large temperature gradient in primary flow as well in secondary flow. Meanwhile, bouncy force has a direct relation versus the pressure gradient which generates the maximum magnitude in MBL (momentum boundary layer thickness). The values of *λ*_1_>0 and *λ*_1_<0 are associated along with the role of cooling and heating processes in fluid particles. In graphical view, cooling process is considered for decrement in flow and heating process is assumed for increasing flow. Moreover, nanoparticles are not enough significant to obtain maximum flow as compared maximum flow for hybrid nanoparticles. Hence, the change in *λ*_1_ becomes favorable physical parameter against temperature gradient and bouncy force. This happening derives the fluid significant over heated surface. Here, dual role of *λ*_1_ is notices against flow. The thickness related (BL) is increased using the higher values of *λ*_1_. The variation in *H*_*s*_ against the primary velocity is conducted by Fig 6. The flow is enhanced using enlargement in *H*_*s*_. The internal heat in fluid particles are increased due to *H*_*s*_ and internal heat has direct relation versus kinetic energy. Hence, direct relation is simulated versus *H*_*s*_. Therefore, MBL become significant. The large values of *H*_*s*_ produces more heat energy into fluid particles. Therefore, heat energy of hybrid nanoparticles and nanoparticles is increased. It is noticed that property related to generative of heat energy is not suitable when hybrid nanoparticles are considered as a coolant.

## 4.2 Graphical outcomes of thermal energy

The measurement of temperature is essential part in this research article. In fact, comparative simulations are captured inserting nano and hybrid nano-structures in base fluid versus *H*_*s*_, Ω_*a*_ and *λ*_1_. In Figs 8–10, it is essential to mention that hybrid nanoparticles are more efficient to obtain maximum enhancement in thermal conductivity rather than the role of nanoparticles. Therefore, hybrid nanoparticles are efficient strong to achieve maximum production of heat energy and cooling process. Fig 8 conducts the distribution of thermal energy against *λ*_1_. The reduction in the production of heat energy is achieved versus the higher *λ*_1_. In physical point of view, temperature gradient enhances due to large values of *λ*_1_ and more acceleration is produced in velocity. Physically, flow of fluid particles accelerates and temperature gradient increases due to higher values of *λ*_1_. These dual influences enhance the flow and thermal energy transfer. Further, These dual influences decrees internal energy of particles. Hence, these happenings create decline in temperature profile. The solid curves are higher than curved related to das dot lines. The graphical study with respect to *H*_*s*_ is addressed by Fig 9. The fluid particles are heated due internal energy source. So, more heat is generated in fluid particles using *H*_*s*_. The large values of *H*_*s*_ produces more heat energy into fluid particles. Therefore, heat energy of hybrid nanoparticles and nanoparticles is increased. It is noticed that external heat source is considered at surface of sheet generate more heat into fluid particles. Hence, fluid particles are heated due to enhancement of heat generation number. Therefore, increasing function is conducted of heat energy. Moreover, maximum amount of heat energy is captured inserting hybrid nanoparticles rather than inserting the nanoparticles. Fig 10 addresses decreasing character of heat energy against the change in Ω_*a*_. It is noticed that Ω_*a*_ is constructed due to non-Fourier’s concept using in energy equation. The role of Ω_*a*_ is not favorable to attain the maximum production in heat energy. The current study is transformed into the case of Fourier’s law for Ω_*a*_ = 0. Finally, the case regarding Fourier’s law is more significant to attain maximum heat energy as compared the non-Fourier’s theory. Here, Ω_*a*_ denotes the phenomena of thermal relaxation. The higher values of thermal relaxation time enhance the performance of fluid related to restore the equilibrium condition. This effect brings minimizing change into the thermal state of fluid particles. Therefore, it has direct effect on TBL (thermal boundary layer thickness).

### 4.3 Outcomes related to gradient temperature and divergent flow

The divergent flow and gradient temperature are noticed versus *We*, *λ*_1_, *H*_*s*_ and Ω_*a*_ simulating in Table 4. The reduction in gradient called skin friction coefficient versus variation in *We* and *λ*_1_ but increasing values of divergent flow is simulated against *We*. Physically, flow of fluid particles accelerates and temperature gradient increases due to higher values of *λ*_1_. These dual influences enhance the flow and thermal energy transfer. Further, These dual influences decrees internal energy of particles. In comparative view, hybrid nanoparticles make the more enhancements in divergent flow as compared nanoparticles. Hence, nanoparticles are more essential rather than study of nanoparticles in view of divergent velocity. The gradient temperature is enhanced versus heat generation number due to gravitational force. But inverse trend is simulated in temperature gradient due to using the large values of Ω_*a*_. Meanwhile, it is observed that study of hybrid nanoparticles make the more impact to attain maximum temperature gradient called Nusselt number rather than the study of nanoparticles.

**Table 4:** Comparative simulations of gradient temperature and skin friction coefficient considering nanoparticles and hybrid nanoparticles versus the change in *We*, *λ*_1_, *H*_*s*_ and Ω_*a*_.

		Nanoparticles		Hybrid nanoparticles	
		−(*R*_*e*_)^1/2^*C*_*f*_	−(*R*_*e*_)^−1/2^*Nu*	−(*R*_*e*_)^1/2^*C*_*f*_	−(*R*_*e*_)^−1/2^*Nu*
	0.0	0.40101802	0.31201902	1.01219001	2.13201732
*We*	0.2	0.36352971	0.44888821	1.20065603	2.55502285
	0.7	0.22859163	0.41301203	1.335083774	2.31001730
	0.7	0.417202388	0.34110303	1.413212063	2.23210163
*λ*_1_	-1.3	0.3082075083	0.44888821	1.510071963	3.55502285
	0.3	0.2082084230	0.53410120	1.310072391	3.73210132
	1.2	0.1082089891	0.61004305	1.210072658	3.91213031
*H*_*s*_	-1.2	0.5082209234	0.77329020	2.710090674	3.27237837
	0.5	0.2101789003	0.401603593	2.231073203	3.008146408
	0.0	0.0083779234	1.3123635	0.010284611	3.3180474
Ω_*a*_	0.5	0.0083779234	1.5012340	0.010284611	3.4231303
	1.5	0.0083779234	1.7092120	0.010284611	3.7121313

### 5. Conclusion and prime findings

The features of magnetic induction in tangent hyperbolic liquid suspending with nanoparticles and hybrid nano-structures is addressed. The role of non-Fourier’s theory along with heat generation is considered. This complex model is simulated with strong technique called finite element approach (FEM). The prime outcomes related to current model is captured below:

- The hybrid nanoparticles are more efficient to conduct maximum production of heat energy as compared the production of heat energy of nanoparticles. Hence, hybrid nanoparticles (*MoS*_2_/*Ag*) are observed more significant to conduct more heat energy rather than nanoparticles (*Ag*);
- The flow is significantly enhanced for the case of hybrid nanomaterials as compared for the case nanomaterials. The flow of hybrid nanoparticles is higher than flow of nanoparticles considering various physical parameters;
- The convergence of current model is analyzed up to 300 elements. The implementation of FEM is considered very useful for discretization of derivatives and this approach handles various types BCs (boundary conditions);
- The power law and Weissenberg numbers indicate the reduction in growth of velocity but flow is enhanced versus the large values of heat generation and mixed convection number. The thickness of boundary layers is declined considering higher values of Weissenberg number while thickness of boundary layers in inclined versus the change in power law number;
- The maximum amount of heat energy is simulated using large value heat generation number but less production of heat energy is attained against the variation of time relaxation and mixed convection numbers. Further, thickness related to thermal layers becomes higher versus the values of heat generation number. The variation of time relaxation and mixed convection numbers make the reduction in thermal boundary layer thickness.
- The temperature gradient is enhanced versus enlargement in time relaxation and mixed convection numbers while decimation in temperature gradient is captured using higher values of heat generation and Weissenberg numbers. Moreover, hybrid nanoparticles are also considered useful to maximized production of temperature gradient rather than the production of temperature gradient for the case of nanoparticles;
- The skin friction is reduced versus enhancement in heat generation mixed convection numbers but reverse role is addressed versus the higher values of Weissenberg and mixed convection numbers. The surface force becomes higher for hybrid nanoparticles than surface force for the case of nanoparticles.
